# Multi-decadal coastal change detection using remote sensing: the Mediterranean coast of Egypt between El-Dabaa and Ras El-Hekma

**DOI:** 10.1007/s10661-024-12359-x

**Published:** 2024-01-22

**Authors:** Esraa A. El-Masry, Asmaa Magdy, Ayman El-Gamal, Baher Mahmoud, Mahmoud Kh. El-Sayed

**Affiliations:** 1 Department of Oceanography, Faculty of Science, Alexandria University, Alexandria, Egypt; 2 Marine Geology Department, Coastal Research Institute, National Water Research Center, Alexandria, Egypt

**Keywords:** Supervised classification, Coastal zone, Change detection, Remote sensing, GIS, Conflict in land use

## Abstract

A key source of information for many decision support systems is identifying land use and land cover (LULC) based on remote sensing data. Land conservation, sustainable development, and water resource management all benefit from the knowledge obtained from detecting changes in land use and land cover. The present study aims to investigate the multi-decadal coastal change detection for Ras El-Hekma and El-Dabaa area along the Mediterranean coast of Egypt, a multi-sectoral development area. Besides, the superiority of the area is highly dependent on its proximity to three development projects: the tourism and urban growth pole at Ras El-Hekma, the beachfront Alamain New Mega City, and the Nuclear Power Plant at El Dabaa. This study utilized multi-spectral Landsat satellite images covering 1990, 2010, and 2020 to perceive the post-classification change detection analysis of the land use and land cover changes (LULCC) over 30 years. The results of the supervised classification from 1990 to 2020 showed a 47.33 km^2^ (4.13%) expansion of the agricultural land area, whereas the bare soil land area shrunk to 73.13 km^2^ (6.24%). On the other hand, the built-up activities in the area launched in 2010 and escalated to 20.51 km^2^(1.77%) in 2020. The change in land use reveals the shift in the economic growth pattern in the last decade toward tourism and urban development. Meanwhile, it indicates that no conflict has yet arisen regarding the land use between the expanded socioeconomic main sectors (i.e., agriculture, and tourism). Therefore, the best practices of land use management and active participation of the stakeholders and the local community should be enhanced to achieve sustainability and avoid future conflicts. An area-specific plan including resource conservation measures and the provision of livelihood alternatives should be formulated within the National Integrated Coastal Zone Management (ICZM) plan with the participation of the main stakeholders and beneficiaries. The findings of the present work may be considered useful for sustainable management and supportive to the decision-making process for the sustainable development of this area.

## Introduction

Coastal zones are constantly changing at different spatial and temporal dimensions around the world. These changes are usually introduced by man-made activities, natural processes, or a combination of both and other factors. The ability of ecosystems to provide their services to societies, both in terms of quantity and quality, is impacted by these changes. Therefore, it is crucial to comprehend how land abandonment, urbanization, and agricultural intensification affect the availability of ecosystem services (Pereira et al., 2022). Parthasarathy and Deka (2021) emphasized that due to the overexploitation and unsustainable use of resources, the rate of change and the vulnerability of the coast should be assessed. Thus, the structure of the environment and the pressures it faces are of prime priority in the investigation of coastal development (Apostolopoulos & Nikolakopoulos, 2021). Sustainability has emerged as a key goal in modern ecosystem management as one of its effects is the ongoing requirement for accurate and current resource data (Chen et al., 2019; Coppin et al., 2004). Recognizing landscape patterns, changes, and how human activities interact with natural phenomena are crucial for effective land management and decision-making (Rawat & Kumar, 2015). Information on land use and land cover (LULC) are crucial for understanding the changes in the coastal system to meet the growing demands for essential human needs and well-being. Change detection is useful in numerous applications for detecting alternation in land use and land cover, such as agriculture, urban growth, and landscape changes (Hegazy & Kaloop, 2015).

Nowadays, the area between El-Dabaa and Ras El-Hekma (study area) is considered a development hub. During the next decade, Egypt conceptualized the Ras El-Hekma Waterfront New City to achieve a qualitative transformation in the spatial planning of new resilient cities (https://unhabitat.org). Moreover, the superiority of the area is highly dependent on its proximity to three development projects: the tourism and urban growth pole at Ras El-Hekma, the beachfront Alamain New Mega City (60 km E to the study area) and El-Dabaa Nuclear Power Plant “NPP” situated inland (about 3.5 km) at about (5.6 km E to the study area). Therefore, spatial planning will enhance the ability of this region to cope with the existing and anticipated pressures from growing population, industrialization, and development of coastal resorts as well as the impacts of climate change.

Currently, satellite data are largely used for (LULCC) assessment studies. Remote Sensing (RS) and Geographical Information System (GIS) techniques are simple and cost-effective tools for comprehending the dynamics of the landscape and provide high-resolution, informative, precise, and up-to-date data and information to investigate (LULCC) in less time, at a lower cost, and with greater precision, they are more effective than traditional tools (Kasereka et al., 2010).

An archive was created in 1972 using multispectral satellite imaging, including its ability to cover almost the whole planet, its vast data record, and the availability of many images taken at different times in the same location (Apostolopoulos & Nikolakopoulos, 2020). Therefore, multispectral satellite imagery may be more effective than other methods in the short- medium- and long term for identifying the trend of coastal change (Almonacid-Caballer et al., 2016). The short-term change reveals the magnitude of the variability over a year due to a specific phenomenon (e.g., a major storm). On the other hand, the medium- and long-term changes are more significant because they support predictions of the foreseen impacts of some cross-cutting issues such as climate change (Pardo-Pascual et al., 2012). In several coastal regions, where a significant tourism sector is established on beach resources, it could be strategically crucial to identify the significance and rate of changes as this would facilitate the implementation of management measures aimed at reducing risk (Dupeyras & Maccallum, 2013). Numerous studies attempted to examine and determine the best approach for (LULCC) by using a variety of computations and algorithms. Moreover, automatic classification techniques were utilized to identify coastal (Teodoro & Gonçalves, 2012; Teodoro et al., 2014)patterns using satellite data and aerial images.

The Landsat images acquired by the TM and ETM + sensors on the Landsat 5, 7, 8, and 9 series are the largest useable database of medium-resolution images for studying the dynamics of coastal areas (Apostolopoulos & Nikolakopoulos, 2020). Landsat data sets are very valuable for large-scale studies because they cover the entire planet continuously for more than 50 years (Apostolopoulos & Nikolakopoulos, 2022). On the other hand, many studies have used high-resolution (0.5–5 m) satellite images. For example, IKONOS-2 imagery has been employed by Pantanahiran (2019) and Kaliraj et al. (2017). Worldview-2 has been used by Pantanahiran (2019) and Vassilakis et al. (2016). RapidEye has been used by Duarte et al. (2018). Corona has been used by Annibale et al. (2009) and Pleiades has been used by Pantanahiran (2019).

According to Apostolopoulos and Nikolakopoulos (2021), satellite images are crucial tool for studying the multitemporal evolution of many different natural phenomena, including coastal ecosystems and shoreline evolution. Its spatial resolution, suitable only for large-magnitude changes, is the main technical issue with using this repository. To investigate the overall evolution of coastal areas, the Landsat archive was thus appropriate (Apostolopoulos & Nikolakopoulos, 2020).

Various methods have been developed to use remotely sensed data to detect the (LULCC), such as supervised classification, clustering or unsupervised classification, hybrid classification Principal Component Analysis (PCA), neural network, and fuzzy classification (Shafique et al., 2022). Various supervised classification techniques are widely used to calculate (LULC) changes all around the world. However, this classification method is highly reliant on subject-matter expertise and familiarity with the area being observed (Butt et al., 2015). Consequently, this method utilizes the signature and digital numbers (DN) of each pixel in the image and converts them into radiance values (Mondejar & Tongco, 2019).

The (LULCC) worldwide has been recently studied and documented in various research. Yagoub and Kolan (2006) assessed and quantified the changes in LULCC that occurred in Abu Dhabi’s coastal zone between 1972 and 2000 to create effective management strategies. Kaliraj et al. (2017) estimated the decadal changes and transformations of (LULC) features along the Kanyakumari coast, of India during the period from 2000 to 2011. Mohajane et al. (2018) described the vegetation change of Azrou Forest in the Middle Atlas, Morocco, between 1987 and 2017. Guidigan et al. (2019) evaluated the LULC dynamic and its effects in Benin Republic, West Africa. Gondwe et al. (2021) identify and quantify (LULCC) that happened in Blantyre City (Malawi) over 20 years. Ngondo et al. (2021) studied the (LULCC) detection over 28 years in the Wami-Ruvu Basin located in Tanzania. Randazzo et al. (2021) investigated the supervised classification methods for the (LULC) mapping of the pocket beaches located in the province of Messina, Italy. Abebe et al. (2022) analyzed the status of (LULCC) 30 years ago in the Gubalafto district in Northeastern Ethiopia. Atayi et al. (2022) evaluated (LULC) along the coastal zone of Keta (Ghana) between 2000 and 2021. Seyam et al. (2023) identified (LULCC) along Mymernsingh, Bangladesh, for the period from 2002 to 2020. All these studies, for example, helped to establish the discipline of change detection based on temporal trajectory analysis, which compares the same area over a longer period using various imageries.

Several studies monitored the land cover changes in the Mediterranean northwestern coastal area of Egypt using remote sensing and (GIS). Shalaby and Tateishi (2007) reported that the land cover along the northwestern coastal zone of Egypt, encompassing the study area, has experienced severe change. Abd El-Kawy et al. (2011) used remote sensing data and GIS to evaluate (LULCC) detection in the western Nile Delta including a part of the northwestern coast of Egypt from 1984 to 2009. Elzahaby et al. (2015) identified (LULC) classes and their change in the Burg El Arab region (SE of the study area) over a period from 1984 to 2014. Halmy et al. (2015) identified (LULCC) in part of the northwestern desert of Egypt for the period 1988, 1999, and 2011 using the Markov-CA integrated approach to predict future changes. Mohamed et al. (2018) used two different classification methods to compare the different (LULC) classes of the landscape of the area located between Al-Alamien and Ras El-Hekma applying the terrain attributes method and the topographic position index (TPI) method.

Although the area between El-Dabaa and Ras El-Hekma is one of the rapidly developed areas along the northwestern coast of Egypt, detailed information on (LULCC) are scarce and not sufficient. To fill this gap in the literature, it was deemed necessary to monitor the (LULCC) on the entire area over an extended considerable period (30 years) for this study. This study will therefore provide information to planners, land managers, and decision-makers that might help in formulating sustainable policy development and support the establishment of an efficient management plan for this area.

Accordingly, the present study aims to analyze the multi-decadal coastal change including the (LULCC) detection using (RS) and (GIS) tools and techniques for the coastal area between El-Dabaa and Ras El-Hekma, Mediterranean Sea, Egypt, to:

- Integrate supervised maximum likelihood classification with a visual interpretation of RS data to produce both contemporary and historical (LULC) maps for the study area.
- Recognize the long-term (30 years) trend of (LULCCs) through tracking changes from multi-temporal Landsat images using post-classification comparison.
- Identify the conflicts on land uses due to the development of the area, and
- Provide information to planners, land managers, and decision-makers for the sustainable management and development of this area.

## Study area

**Fig. 1:**
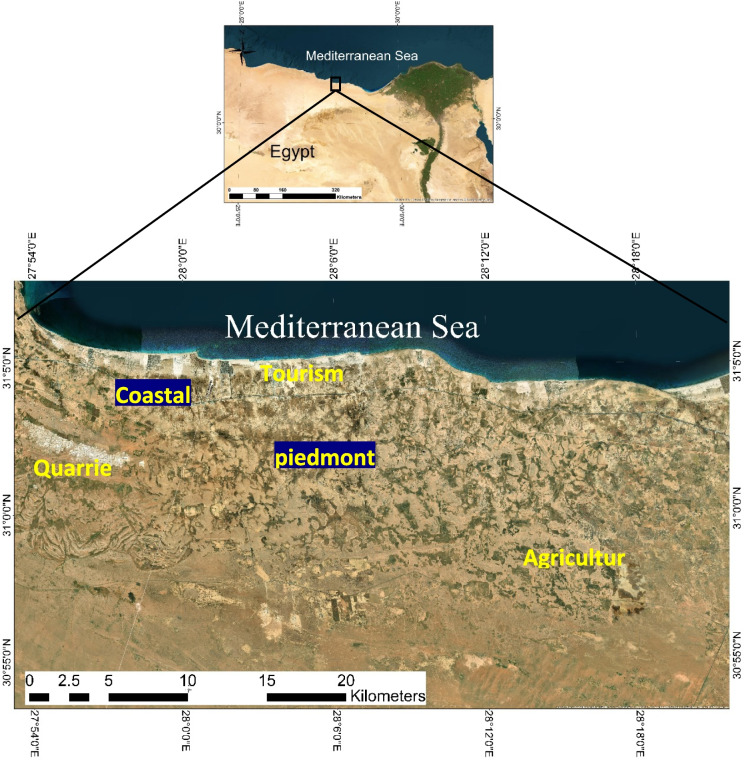
Location map of the study area

The study area is located along the northwestern coastal zone of Egypt between Ras El-Hekma (W) and El-Dabaa (E). It extends for about 141 km, attaining an average width of about 30 km between latitudes 31° 7′–30° 53′ N and longitudes 27° 54′–28° 23′ E, and covering an area of about 1158.5 km^2^ (Fig. 1). About 38,320 inhabitants live in 14 villages in this area (IDSC, 2020).

This area represents a potential hub for future urban and tourism development and expansion as indicated in the Egyptian National Spatial Development Plan “NSDP 2050” and the Integrated Coastal Area Management (ICAM) planning for Fuka-Matrouh (EEAA (Egypt Environmental Affairs Agency), 1996).

The superiority of the area is highly dependent on its proximity to three development projects: the tourism and urban growth pole at Ras El-Hekma, the beachfront Alamain New Mega City (60 km E to the study area), and the inland (about 3.5 km) El-Dabaa Nuclear Power Plant “NPP” situated at about (5.6 km E to the study area). It is anticipated that the study area will not be impacted by the construction and operational activities of these projects since the NW waves generate most of the energy that directly affects the northwestern coast of Egypt (Frihy, 2009). A few years ago, this area witnessed a remarkable increase in tourism activities along its coastal strip. In the hinterland, rural communities were situated for long periods where their main activities were agriculture and pasturing.

Geomorphologically, the study area is divided into three main distinct geomorphic units (from south to north); as follows: The first unit, is a tableland in the south which is made of 150-m-thick Miocene limestone rock. It is covered by thin layers of quartz and weathered carbonate sand and develops into a hard pink crust with many solution holes brought on by precipitation and humidity (Eissa et al., 2018). The main watershed is also being formed at a maximum ground elevation of roughly 200 m above sea level (Yousif & Bubenzer, 2012). The tableland is dissected by many depressions forming basins draining from the south to the north direction toward the Mediterranean. The Piedmont Plain (the second unit) comprises a number of inland limestone ridges separated by depressions (Yousif & Bubenzer, 2012). The coastal plain (the third unit) skirts the Mediterranean coast and is surrounded by sand dunes. Most of the newly constructed coastal resorts and villages were built on top and sides of the coastal limestone ridges.

Geologically, the study area is underlain by sedimentary rocks ranging in age from the Middle Miocene to the Quaternary (Gomaa & Omar, 2009). Middle Miocene deposits cover most of the area and are characterized by shallow marine fissured limestone sediments (Gomaa & Omar, 2009; Yousif et al., 2015). This fissured limestone is considered the sole aquifer in the area containing groundwater under perched conditions (Yousif & Bubenzer, 2012). The Quaternary deposits are related to Pleistocene and Holocene times. Pleistocene limestone deposits have a wide distribution in the area, while Holocene deposits are represented by a variety of unconsolidated deposits, differentiated into alluvial, eolian, and beach deposits (Gomaa & Omar, 2009).

## Methodology

**Fig. 2:**
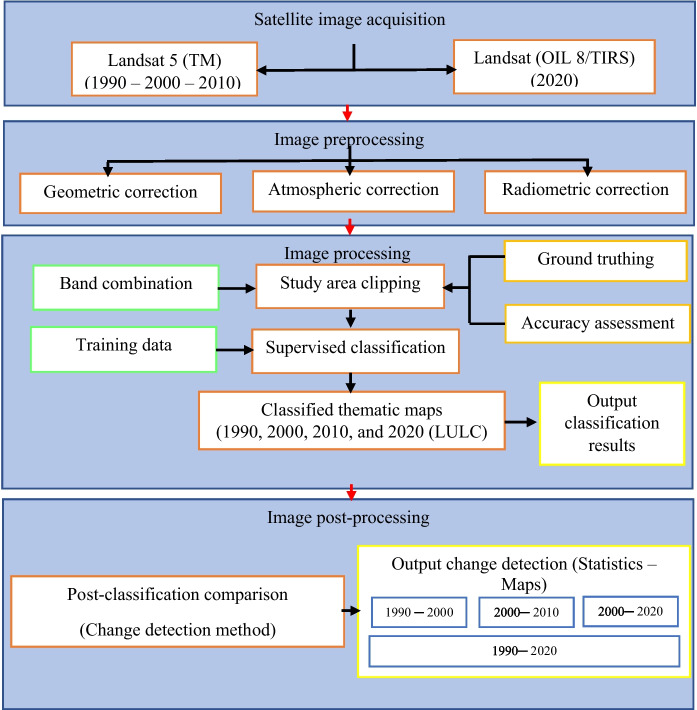
Flowchart of the methodological framework

This study followed two main steps according to Yagoub and Kolan (2006) and El-Hattab ( 2016): The first is the classification of satellite data for (LULC) classes, while the second focused on the change detection analysis in the (LULC) classes. The satellite data analysis is presented. Flowchart of the methodological framework is presented in Fig. 2.

### Data source and pre-processing

Historical Landsat images over 30 years period (1990–2020) were used for studying the historical (LULC) classes. Four Landsat scenes for Path/Row 179/38 from two types of sensors were downloaded from the United States Geological Survey website (http://earthexplorer.usgs.gov/). Table 1 summarizes the characteristics of the Landsat images used in this study.

**Table 1:** Characteristics of satellite imagery used in the present study

Satellite image	Image date	Number of bands	Spatial resolution
Landsat 5 (TM)	21/05/1990	7	30 m
Landsat 5 (TM)	16/05/2000	7	30 m
Landsat 5 (TM)	28/05/2010	7	30 m
Landsat 8 (OLI/TIRS)	07/05/2020	11	30 m

Images were selected in May (spring season) when the sky is usually clear and cloud-free imagery was obtained with the best visibility. This will optimize the comparability and further examine several images in different periods of the year. The image of Landsat 5 (for the year 1990) was used as the “baseline image.” This image shows no coastal development activities at that time. The image of Landsat 5 acquired in 2010 is used as the “reference image” showing the initiation of urbanized activities including coastal resorts.

Before carrying out the image classification step, the raw data followed the standard pre-processing and correction to remove errors caused by atmospheric, radiometric, and earth geometry factors. By employing the Dark object Subtraction (DoS) method, these corrections were made to prevent the errors that are invariably present in satellite images (Mitra et al., 2017).

Layer stacking was performed to make a false-color composite through band combination (combining all bands in a single layer). Ground control points were gathered during the ground truthing in 2020 to correct the 2020 image. Google Earth images from 1990, 2000, 2010, and 2020 were used to establish ten ground control points, the majority of which were significant landmarks. In ArcMap 10.3, the 4 images were co-registered. Each image was further cropped to incorporate the study area specifically using the Region of Interest (ROI) cropping method (Ha et al., 2022). These four cropped images were re-projected to a common projection: Universal Transverse Macerator (UTM) with WGS84 datum and Zone 35 North. Finally, the new layer is ready for display and analysis.

### Supervised classification

The Environment for Visualizing Images (ENVI) version 5.3 software was used to perform supervised classification to estimate the geomorphological features in the study area and prepare the (LULC) maps respectively. There are two types of image classification procedures: region-based and pixel-based. In the present study, pixel-based methods are used for image classification (Teodoro & Gonçalves, 2012). Manfré et al. (2016) identified three algorithms that were tested for supervised classification: minimum distance, maximum likelihood, and parallelepiped with maximum likelihood as a tie-breaker. The spectral information is used to classify each pixel in an unsupervised or supervised pixel-based classification. In addition, the maximum likelihood classification is the most popular supervised classification technique applied with remote sensing picture data based on Bayesian probability theory (Teodoro et al., 2011). Before classifying, the training site and class signature quality must be assessed using the signature separately function (Teodoro et al., 2011). Accordingly, pixels were sorted into a set number of classes based on the brightness value of each pixel. All pixels were automatically categorized in an image, into land cover classes. Selection of the (LULC) training data is the key to supervised classification. Consequently, image processing software is used to calculate the statistical parameters for each information class. Supervised classification was done by Maximum Likelihood Classifier processing (MLC). The (MLC) is one of the most accurate classifiers since it quantitatively assesses the variance and covariance of the category spectral response patterns when categorizing an unknown pixel (Shalaby & Tateishi, 2007). Statistics (area and percentage for each class) have been calculated for each of the identified images and then presented in bar graphs and pie charts the number of pixels and the percentage were calculated for each class separately, and as a result, the area of each class was estimated in terms of km^2^.

### Land-use/land cover change detection

Classification comparisons of land cover statistics were utilized according to El-Hattab (2016). Four change maps for 1990–2000, 2000–2010, 2010–2020, and 1990–2020 were prepared by post-classification comparison method using ENVI (5.3) by running two classification images for the same scene at a different date. In addition, four figures showing the class transition from one class to another were prepared. These transitions were quantified in terms of area in km^2^.

A statistical technique is used to compile a detailed tabulation of changes between the two classification images. Then, (ENVI) thematic change workflow tool was used to measure the transition dynamics of the land cover class to another one at a given extent, the output was then presented in bar graphs and pie charts.

### Accuracy analysis

This study used the Semi-Automatic Classification Plugin (SCP) in (QGIS) software to measure accuracy. Accordingly, a classification error matrix was created by tabulating the reference and classified immaturity data. The components that were common to precision valuation, such as overall accuracy, producer’s accuracy, user’s accuracy, and kappa statistics, were calculated (Kullo et al., 2021). For the accuracy assessment, a total of 70 ground truth points were generated using the stratified random sampling method over the study area for 2020. Out of these reference sites are the Mediterranean Sea (10), bare-soil (20), bare-land/quarries (20), vegetation (10), and built-up (10). These reference sites were then compared to classified results created from the satellite images.

### Limitations

One of the limitations of the present study is the coarse spatial resolutions (around 30 m) of the Landsat satellite images that do not provide enough detailed information to precisely delineate the different classes in a complex urban landscape with mixed pixels as previously mentioned by Weng et al., (2004). To overcome this limitation, the present study adopted a combination of field map-based, aerial photographs, and image classification methods that can be used to reach the sub-pixel details (Esch et al., 2009, and Mountrakis & Luo, 2011).

## Results

### Spatiotemporal analysis of 1990, 2000, 2010, and 2020 satellite images

**Fig. 3:**
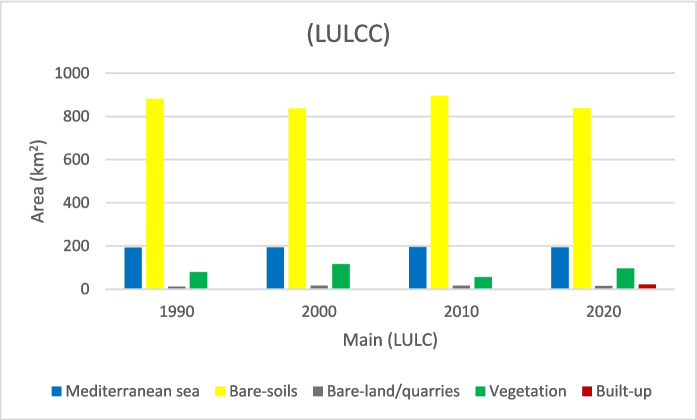
Land use/land cover change of the main sectors of the study area for the years 1990, 2000, 2010, and 2020

The spatiotemporal analysis was carried out to describe trends in land cover and land use change over time. The (LULCC) of the study area images were classified into five main classes as follows: the sea “Mediterranean,” bare-soil, bare-land/quarries, agriculture, and built-up (Fig. 3).

**Fig. 4:**
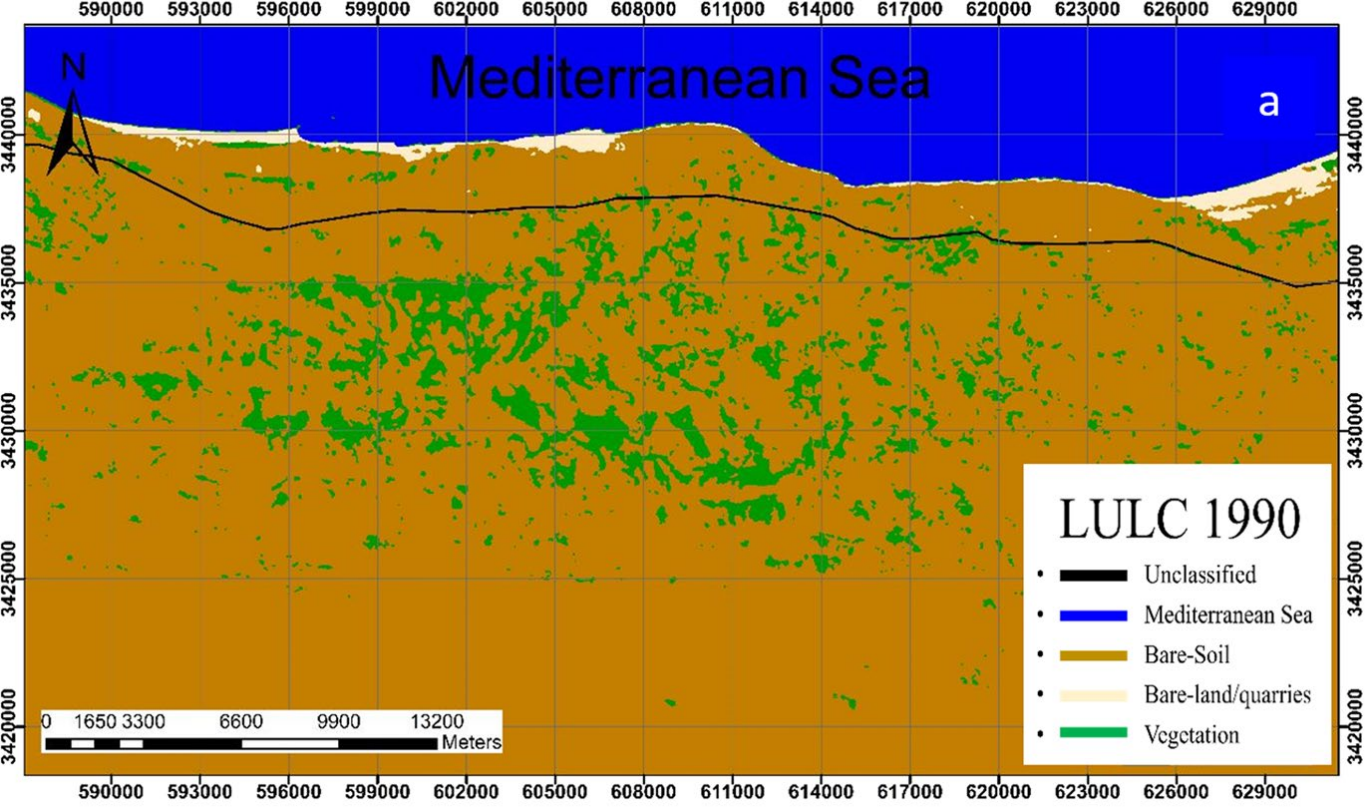
Classified land cover maps of the study area for the year 1990

**Fig. 5:**
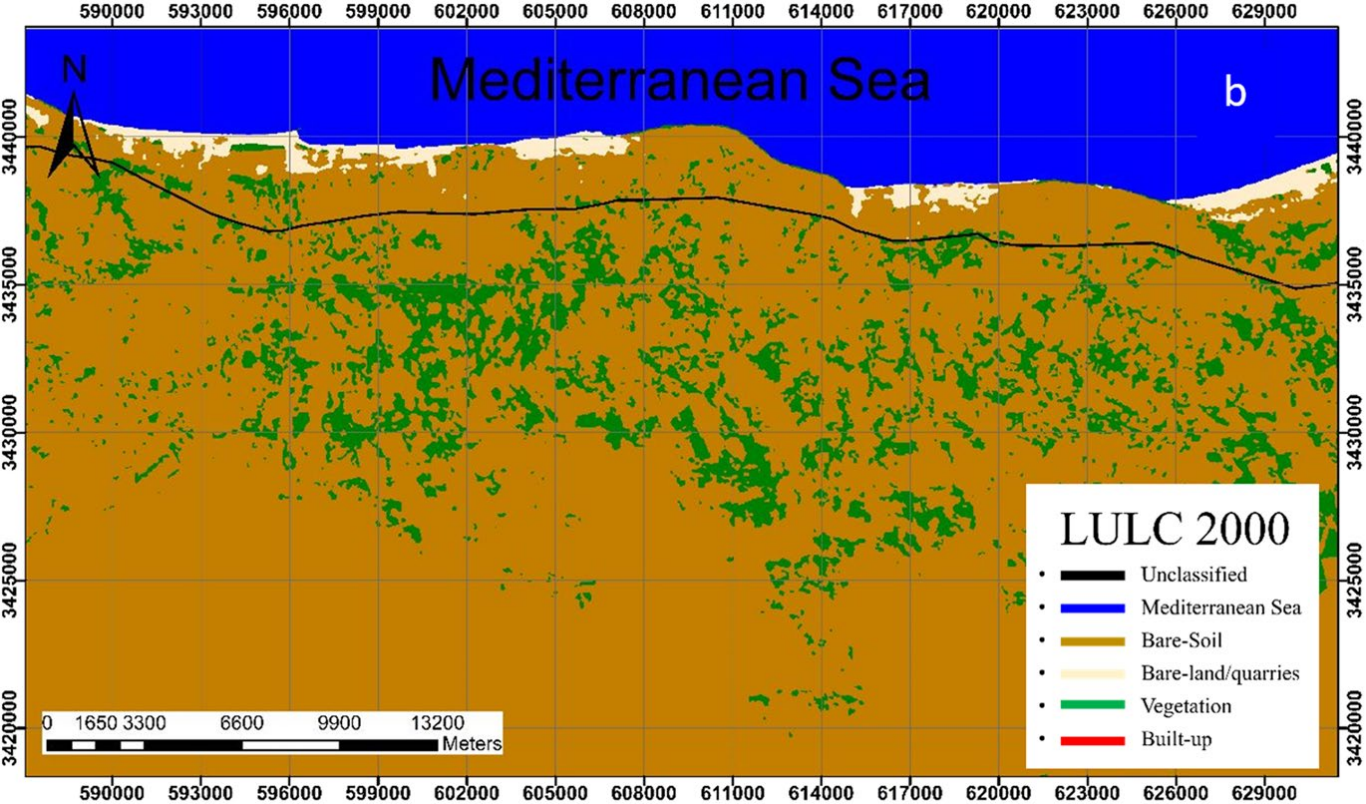
Classified land cover maps of the study area for the year 2000

**Fig. 6:**
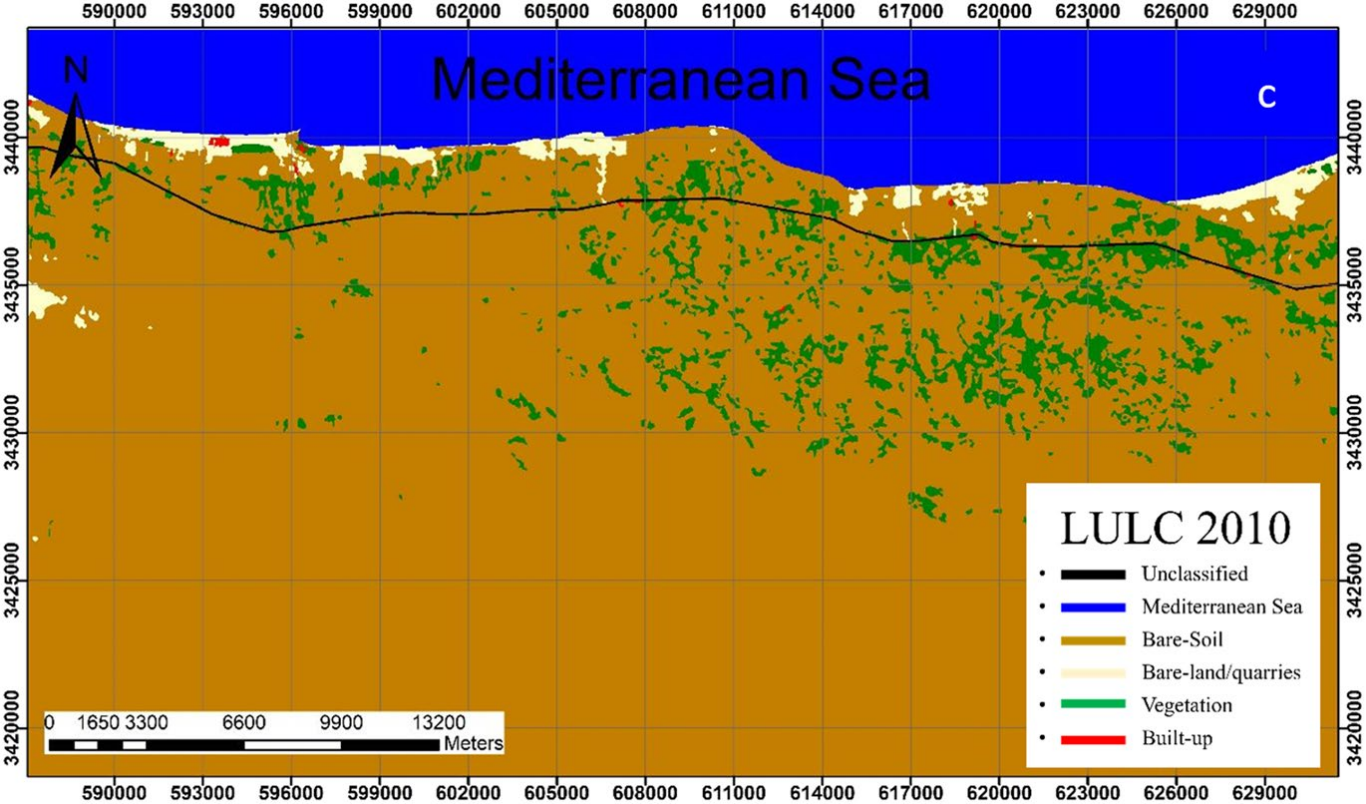
Classified land cover maps of the study area for the year 2010

**Fig. 7:**
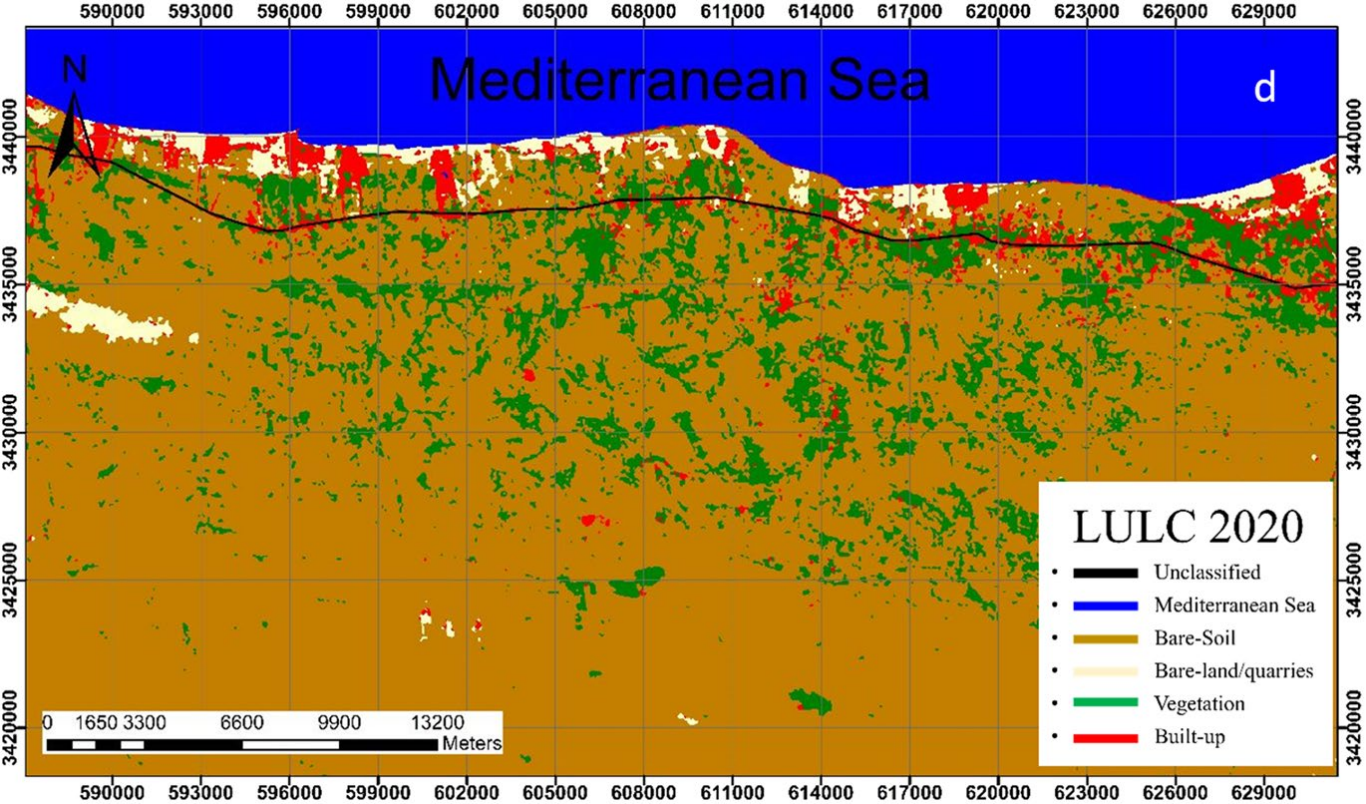
Classified land cover maps of the study area for the year 2020

Analysis revealed that the bare-soil class is the dominant class in the study area. This class shows a slight change throughout the study period where it covers 75.76%, 72.01%, 77.01%, and 72.06% of the total area in 1990, 2000, 2010, and 2020; respectively (Figs. 4, 5, 6, 7).

The variations in vegetation land cover (exposed agricultural lands, cropland, and cultivated areas) generate mixed signatures that imped the classification process. The vegetation area is dominated by rain-fed and groundwater-dependent crops such as figs, olive, barley, and wheat as well as other crops of palm trees, watermelon, and tomato. An increase in vegetated land area in 2000 is observed as the area changed from 78.12 km^2^ “1 km^2^ = 100 hectares” (6.62% from the total study area) in 1990 to about 115.45 km^2^ (9.93% of the total area) in 2000. This finding indicates the expansion of the agricultural land that represents the main and probably the only source of income and welfare for the people living in this rural area at this time. However, with the launching then flourishing of the urban and tourism development in the area in 2010, the vegetated land area shrank to about 55.72 km^2^ (4.7%), i.e., about half its original size in 2000. This marks the shift of the development and economic patterns in the area toward the newly established urban and tourism settlements. This pattern draws higher resources, employment, and investment compared to the dominant traditional rural activities. This net shift in land use revealed that about 95.73 km^2^ of agricultural land has been degraded from 2000 to 2010. The rural and suburban areas have witnessed the growth and flourishing of tourism activities; the local community experiences the benefits of combining both tourism activities in addition to agricultural activities, and in the maturity stages of tourism growth. However, this can cause the choice of one instead of the other (Ghadami et al., 2022). It is worth mentioning that the expansion of the urban and tourism development in the area (2000–2010) has been marginally experienced at the expense of the decline of agricultural land. The conversion of agricultural land (about 83.07 km^2^) to bare soil was probably due to the drought and the lack of water for irrigation.

In 1990, the bare soils in their pristine state were the dominant land class followed by vegetation representing 75.76%, and 6.72% respectively of the total area (Fig. 4). Bare-land/quarries area was about 11.2 km^2^ (0.95% of the total land area) at that time and increased to 16.4 km^2^ (1.41%) in 2000–2010; then further slightly decrease to 14.5 km^2^ (1.25%) in 2020. The reduction of the bare-land/quarries area, particularly in 2000–2020, is attributed to the uncontrolled mining of limestone from this area and the increase in built-up, road construction, and other activities.

The built-up areas that began in 2010 were limited to 1.11 km^2^ (0.1%) of the total coastal area, then it significantly increased to 21.37 km^2^ (1.84%) in 2020, indicating the expansion of infrastructure in this area which is linked to population growth and economic diversity. It is worth noting that the spatial increase of built-up class was mainly at the expense of the bare soil (about 0.65 km^2^).

A net erosion pattern of the coast of the study area during the period of (2010–2020) amounts to − 0.64 km^2^. This erosion is credited to the turbulence of the sediment transport accelerated by the anthropogenic influence associated with coastal urban development. This finding is agreeable with Farghaly (2017) who mentioned that this area was under an erosional state (1984–2016) due to the rapid urban development parallel to the coastline. Other Mediterranean countries including, Algeria, and Moroccan have pronounced coastal erosional patterns. According to Mezouar and Ciortan (2019), the establishment of two new ports in Algeria since 1970 has caused the erosion rate to increase and disrupt the region’s sediment flow (average erosion, -0.24 km^2^). Over 60 years (1958–2018), Benkhattab et al. (2020) examined coastal displacement along the Moroccan coast and reported an average erosion of -0.2 km^2^. Their findings showed that a variety of human activities, such as the continued construction of tourism infrastructure including hotels and ports, a road system along the coast, and sand mining (which obliterated 95% of the coastal dunes) were to blame for the rapid erosion that began in 1986. Additionally, the construction of dams negatively impacted coastal dynamics by drastically decreasing the sedimentary resources. However, Bouchahma and Yan (2012) found that the change in the shore along the Tunisian island of Djerba’s coast from 1984 to 2009 was about -1.7 km^2^. They revealed that the erosional tendency in this area was caused by the disintegration of sand dunes along the coast as a result of the rise in tourism development.

### Accuracy assessment

A dataset’s agreement between two sets of categorizations was estimated using the Kappa statistic (Elsaid Adlan Abdelkareem et al., 2018; Table 2). Data for the years 1990, 2000, 2010, and 2020 showed that the accuracy of the “producer” extends from 60% (2000, 2010, and 2020) for the vegetation class to 100% (2010, and 2020) for the Mediterranean Sea class whereas that of the “user” ranges from 60% (2000) for vegetation class to 100% (1990, and 2010) for the Mediterranean Sea, and vegetation classes, respectively. The overall accuracy ranged from 64.8% (1990) to 85.26% (2010) while the Kappa’s coefficient varied between 0.75 (2000) and 0.85 (1990).

**Table 2:** Accuracy assessment of classification

(LULC)	User’s accuracy (%)				Producer’s accuracy (%)			
	1990	2000	2010	2020	1990	2000	2010	2020
Mediterranean Sea	100	81.81	90.90	90.90	80	90	100	100
Bare-soil	94.11	88.23	89.47	90	80	75	85	90
Bare-land/quarries	71.42	68.18	77.27	80	75	75	85	80
Vegetation	77.77	60	100	85.71	70	60	60	60
Built-up	─	80	75	75	─	80	90	90
Overall accuracy	64.8	75.82	85.26	84.16				
Kappa coefficient	0.85	0. 75	0.86	0.84				

### Change detection analysis

**Fig. 8:**
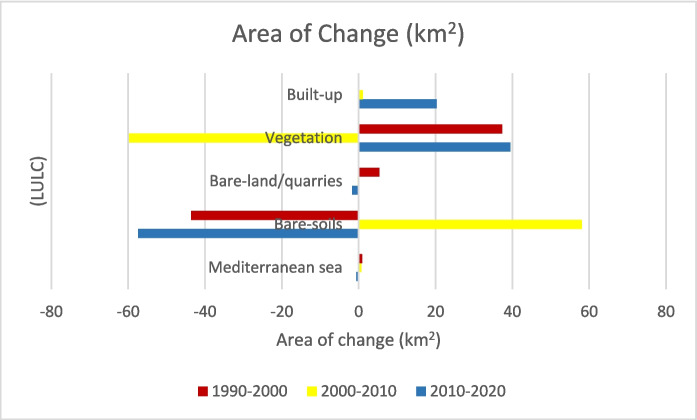
Area of change (km.^2^) of the main (LULC) for the periods (1990–2000, 2000–2010, and 2010–2020)

The change detection analysis is used to create an overall change image for each land cover class during the three periods (1990–2000, 2000–2010, and 2010–2020; Fig. 8). A detailed explanation of the LULC changes for the set periods is discussed below.

#### (LULC) change (1990–2000)

**Fig. 9:**
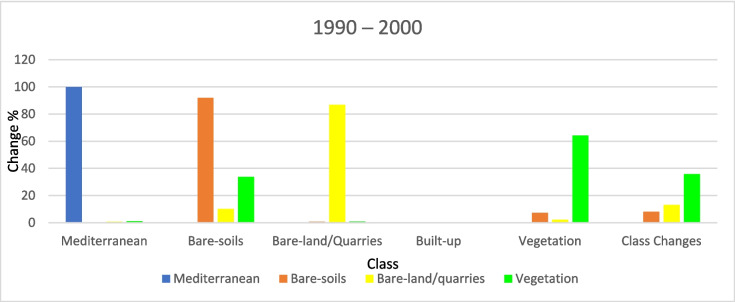
The change percentage of the study area from 1990 to 2000

**Fig. 10:**
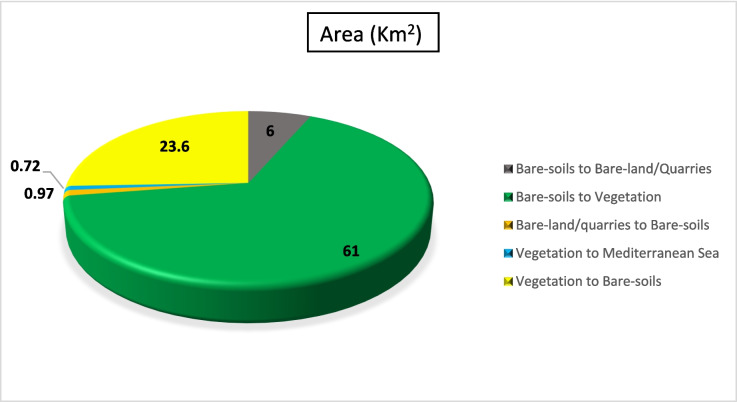
Areas of major classes change statistics (km^2^) for the intermediate period (1990–2000)

**Fig. 11:**
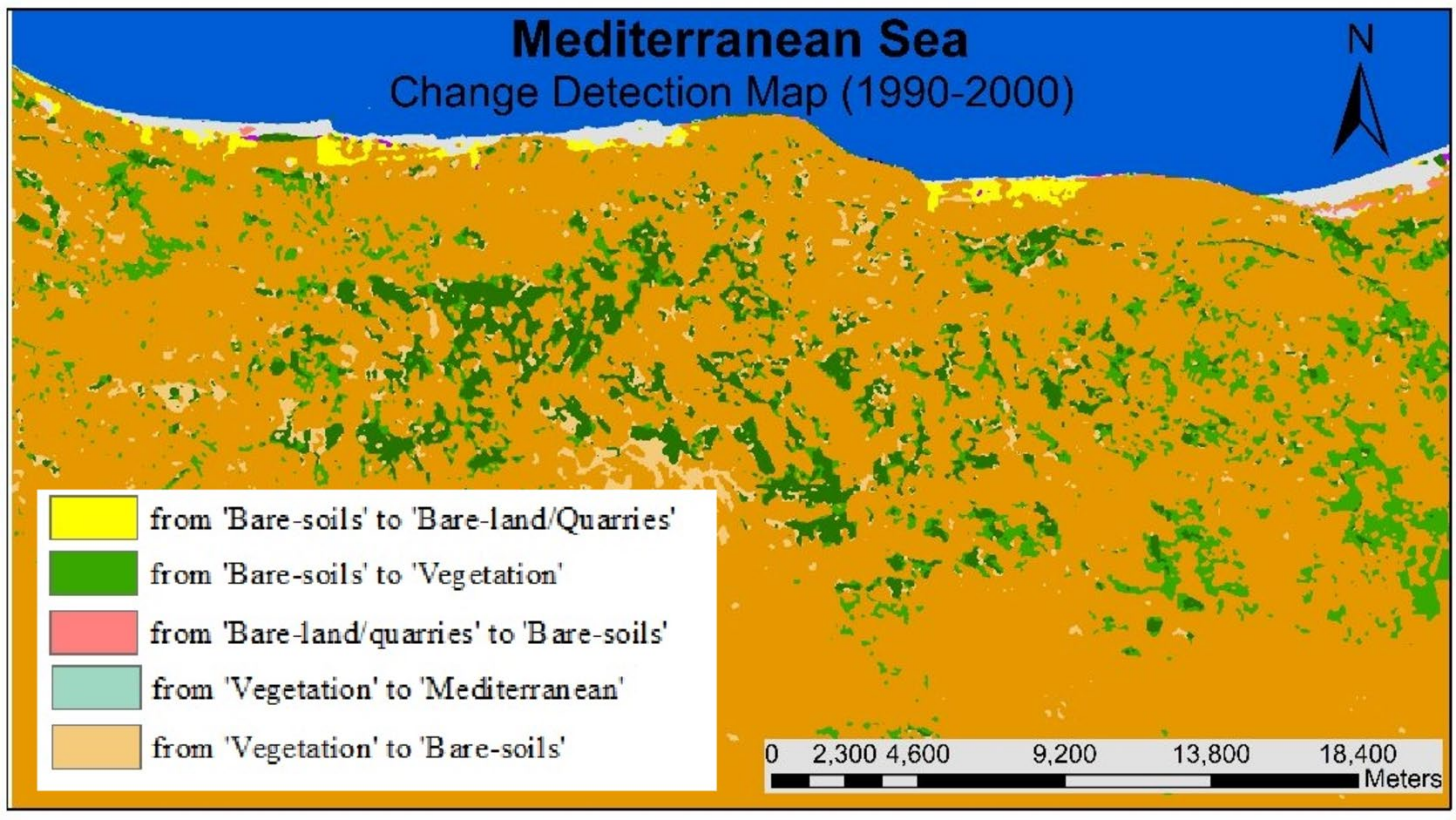
Thematic change workflow analysis for the intermediate period (1990–2000)

Figure 9 shows that the agricultural land areas increased from 1990 to 2000, accounting for approximately 37 km^2^ (about 3% of the study area) while the extent of bare-land/quarries increased by 6 km^2^ (about 0.52%). Figures 10 and 11 present the thematic change statistics and map, including the highest amount of land cover conversion from bare soils to vegetation (about 5%) in (1990–2000). Meanwhile, the same period manifests a minor change from bare soils to bare land/quarries areas (about 2%), while changes in other classes were insignificant.

#### (LULCC) (2000–2010)

**Fig. 12:**
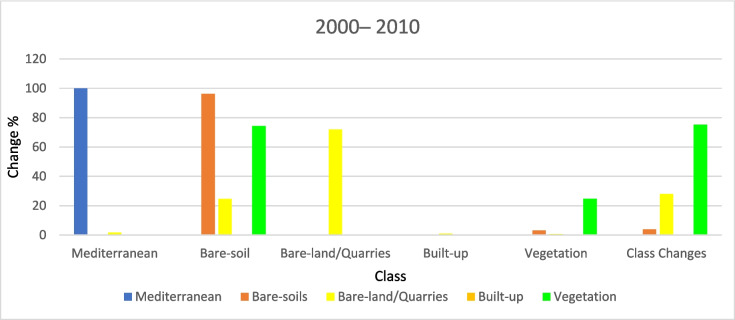
The change percentage of (LULCC) in the study area from 2000 to 2010

**Fig. 13:**
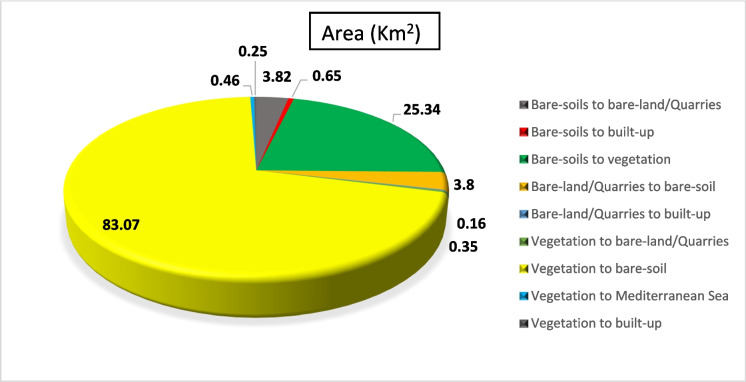
Areas of major classes change statistics (km.^2^) for the intermediate period (2000–2010)

**Fig. 14:**
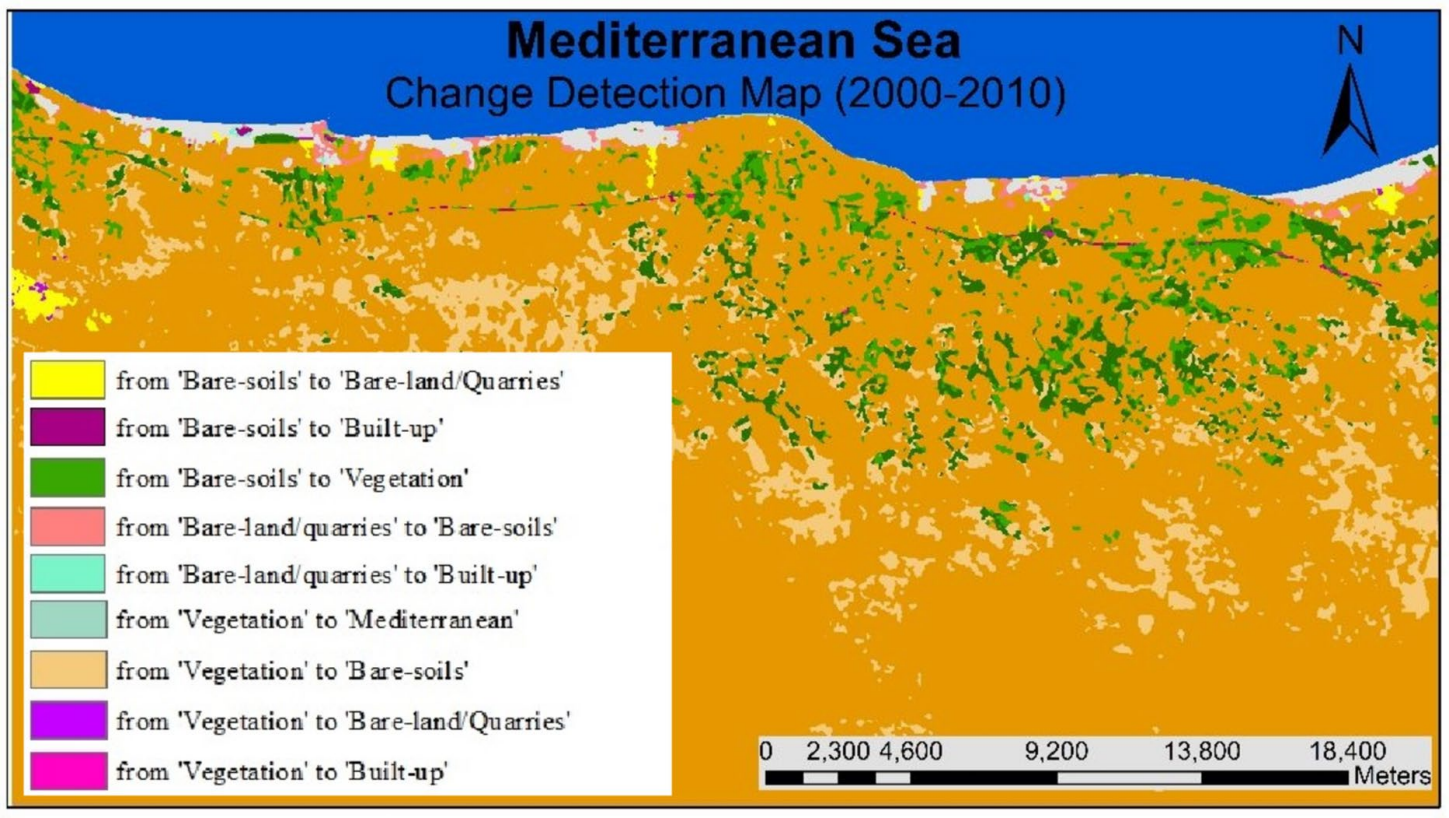
Thematic change workflow analysis for the intermediate period (2000–2010)

Figures 12, 13, and 14 show a drastic conversion (about 74%) from agricultural land area to bare-soil during May 2000– 2010. This change is linked to crop harvesting time during this period since the satellite image was taken in May 2010. Agricultural land was expanded in this particular area during the period (2000–2010) by about 25.3 km^2^. In addition, around 24.5% of the bare-land/quarry area in 2000 was converted into bare soil in 2010. Results of thematic change statistics illustrate that the highest amount of land cover conversion was from vegetation to bare soil (7%), while the smallest amount (about 2%) was from bare soil (2000) to vegetation (2010) (Fig. 13).

#### (LULCC) (2010–2020)

**Fig. 15:**
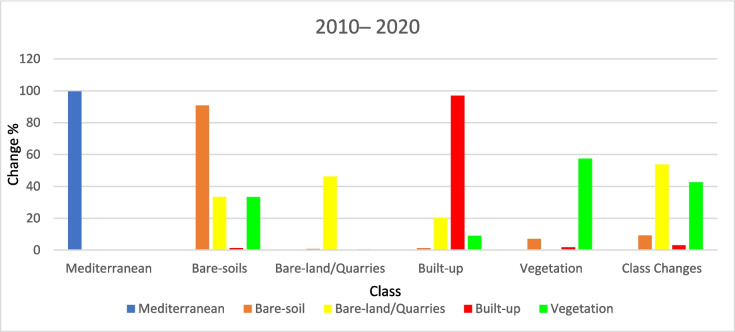
The change percentage of the study area from 2000 to 2010

**Fig. 16:**
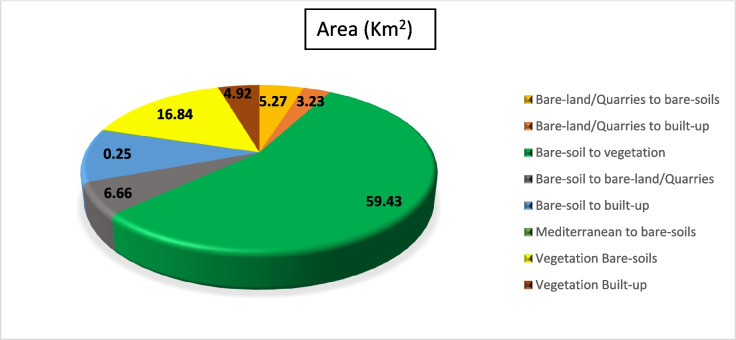
Areas of major classes change statistics (km.^2^) for the intermediate period (2010–2020)

**Fig. 17:**
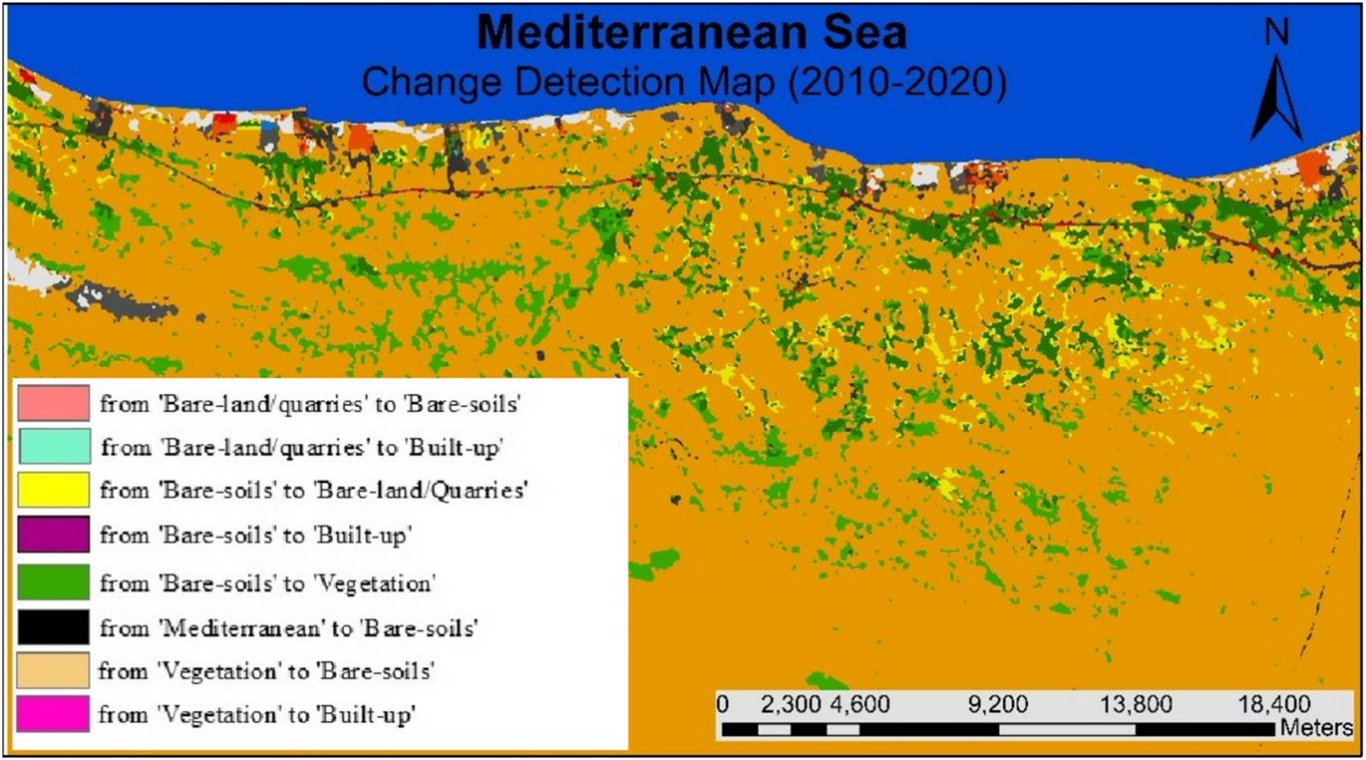
Thematic change workflow analysis for the intermediate period (2010–2020)

**Fig. 18:**
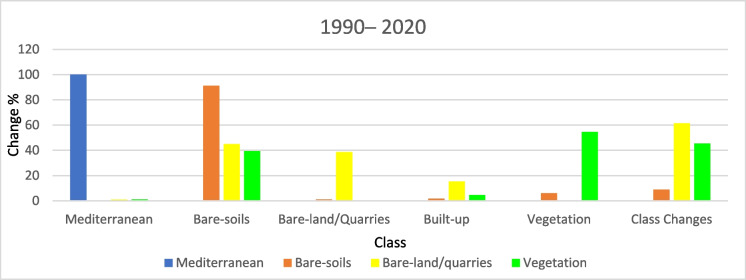
The change percentage of the study area from 1990 to 2020

During 2010– 2020, about 42% of the existing vegetation area in 2010 was converted mainly into bare soil (about 33%) and built-up areas (about 8.8%) (Figs. 15, 16, 17, 18). Meanwhile, around 9% of the bare soil area in 2010 was converted into vegetation area (nearly 7%) and built-up area (about 1.3%). About 53.7% of the bare-and/quarry area in 2010 was converted into bare-soil areas (around 33.3%) and built-up areas (nearly 20%) during the same period. Results of thematic change show that the main land cover conversion was from bare soil to vegetation (approximately 5%.). The minimum recorded change from vegetation to bare soils is about 1.5%.

#### Overall historical (LULCC) (1990–2020)

Quantifying changes in a location over time is the goal of change detection analysis. (Liping et al., 2018). The classified images of 1990, 2000, 2010, and 2020 were utilized to assess the areas and variations of all LULC classes at the starting from 1990 to 2020. The subsequent classified images are also helpful in identifying several changes in all types of land use.

**Fig. 19:**
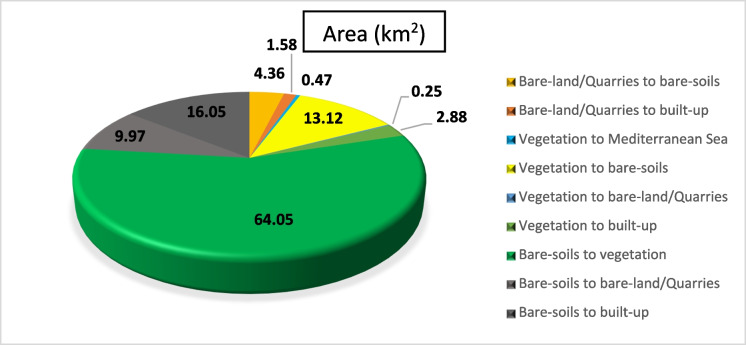
Areas of major classes change statistics (km.^2^) for the period (1990–2020)

**Fig. 20:**
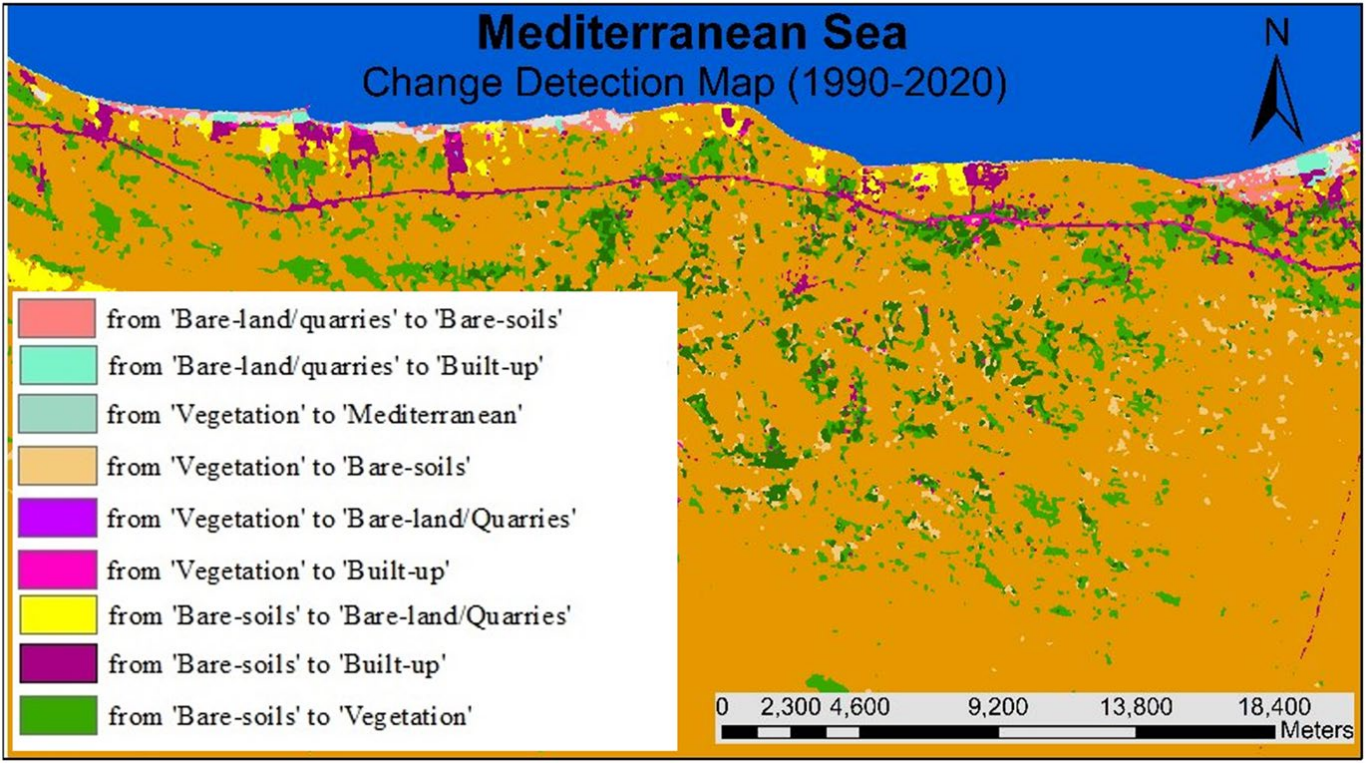
Thematic change workflow analysis for the intermediate period (1990–2020)

The activities related to vegetation, urban development, and quarrying are the key drivers of changes in (LULC) in the study area. Results revealed an overall change of about 113 km^2^ of the (LULC) during the overall study period, i.e., 1990–2020 (Figs. 18, 19, 20). Most of the vegetation class area increased (about 39 km^2^) due to the conversion from bare soil to vegetation. Approximately 20.51 km^2^ of the built-up areas were registered during the overall period. These areas have been acquired from bare-soil (16.05 km^2^) followed by vegetation (2.88 km^2^), and last from bare-land/quarries (1.58 km^2^). The built-up development matches the coastline of the coastal plain area, while the agricultural expansion is linked to the Piedmont plain area (hinterland) (Fig. 20).

Most of the land uses have been diverted into urbanization, due to land speculation, population growth, and rapid economic development. As a hub of tourism development, the coastal zone between El-Dabaa and Ras El-Hekma ranked at the top of the most developed regions of the northwestern coast of Egypt and will presumably maintain a high level of economic development in the upcoming years. This region has drawn a significant number of investors and settlers from other parts of the country as well as from abroad due to the economic growth, and environment quality. As a result, substantial areas of agriculture have been encroached upon for urban sprawl, including for real estate and agro-industrial purposes.

## Discussion

Fortunately, the study area as such is not yet influenced by remarkable socio-economic conflicts of land uses in both agricultural and tourism sectors and other development sectors. However, anticipated conflicts may later occur due to the rapid growth of the tourism sector and urbanization in the area at the expense of other development sectors. Busby and Rendle (2000) noted that in some cases, the conflict between the agriculture and tourism sectors is such that even if tourism grows in the form of agro-tourism.

Based on the study findings in the context of regional coastal zone issues, rapid industrialization and urbanization have led to a decline in vegetation, while built-up areas have increased due to settlement and infrastructural improvements. The most significant change is seen in built-up areas, with increased agriculture and settlements leading to a decrease in grazing and forest lands. This is attributed to population growth, unpredictability of land tenure, climate change, and lack of public awareness.

Yagoub and Kolan (2006) recognized that rapid urbanization along the Abu Dhabi coastal zone between 1972 and 2000 caused a decrease in woody vegetation areas. In addition, they reported that to preserve these significant coastal zone land use types before they become extinct, sound land use management measures must be developed. All countries prioritize coastal zone conservation, so it is necessary to build and integrate a spatial and non-spatial database for the coastal zone to improve management and decision-making procedures. Kaliraj et al. (2017) illustrated that human encroachment activities result in the conversion of cultivable lands into built-up areas, whereas natural processes like land degradation and surface runoff transform cultivable fields into barren areas. The study by Guidigan et al. (2019) along Benin Republic (West Africa) portrays a worrying scenario showing a significant decrease in Savannah land and bare lands due to the rapid expansion of built-up area and a rise in human activities and crop production. It also raises awareness of the need for sustainable landscape development and management. Gondwe et al. (2021) found that the built-up and agricultural lands increased while bare land and forest lands decreased in Blantyre City (Malawi)**.** Ngondo et al. (2021) noticed that a decrease in water, wetland, and forest areas was replaced by an increase in agricultural, bare soil, and built-up areas along the Wami-Ruvu Basin located in Tanzania. The findings of Abebe et al. (2022) showed increasing agriculture and settlement and a decrease in grazing and forest lands in the Gubalafto district in Northeastern Ethiopia. They noted that the population growth, unpredictability of land tenure and common property rights, climate change, and a lack of public awareness are the main factors causing (LULCC) in the area. They suggested that (LULCC) causes must be controlled, and usage of sustainable resources is crucial; otherwise, these limited natural resource bases would rapidly disappear and lose their ability to support sustainable ecosystem services. Atayi et al., (2022) assumed that rapid urbanization and agricultural activities are to blame for the decline in the closed forest cover in the Keta (Ghana). Seyam et al. (2023) found that the built-up area experienced the most change as a result of urbanization and industrialization which has a substantial impact in Mymernsingh (Bangladesh).

In related studies regarding the land use/land cover changes (LULCC) in the Mediterranean northwestern coast of Egypt, Shalaby and Tateishi (2007) reported that the land cover has experienced severe change accordingly to development projects such as tourism or agriculture, as a remarkable development in urbanized areas. Abd El-Kawy et al. (2011) evaluated the (LULCC) in part of this area from 1984 to 2009 andreported major changes from bare land to agricultural land. Elzahaby et al. (2015) identified (LULC) classes in the Burg El Arab region (SE of the present study area) over a period from 1984 to 2014; they found that increasing in urban and agricultural lands and decreasing the bare lands reflected the change in reclamation and urbanization and industrialization activities. (LULCC) study in part of the northwestern desert of Egypt for the period 1988, 1999, and 2011 demonstrates that the irrigation network became operational, irrigated agriculture grew to the detriment of orchards and other types of rain-fed agriculture (Halmy et al., 2015). Mohamed et al. (2018) pointed out that the migration from rural to urban areas, highway ribbon construction, and effective transportation infrastructure have all been significant change-causing factors in the conflict Al-Alamien and Ras El-Hekma sectors. Most of these findings concurred with main results and outcomes of the present study.

The relationship between tourism and agriculture is weakening in several destinations around the world. Therefore, the growth of tourism activities has led to a decrease in agricultural lands and a reduced agricultural labor force (Fleischer & Tchetchik, 2005). Ghadami et al., (2022) confirmed the above by stating that in rural and suburban areas, which face the growth of tourism activities, the local community experiences the benefits of tourism activities in addition to agricultural activities, and in the maturity stages of tourism growth, this can cause the choose of one instead of the other.

## Conclusions

The (LULC) alterations in the study area have undoubtedly been impacted by the land reclamation initiatives in Egypt carried out during the past three decades. Accordingly, areas used for agriculture have expanded significantly and tourism activities have also rapidly increased. These(LULCCs) experienced in local policy and the dynamics of human impacts on the study area, have enhanced agricultural production, which has created jobs and greatly increased population density in the former agricultural areas.

However, the area of study is not yet influenced by remarkable socio-economic conflicts of land use in both agricultural and tourism sectors due to the changes in the land use pattern, however, anticipated conflicts are expected due to the growth of the tourism sector and urbanization in the area on the expenses of other land classes. However, it is worth mentioning that with the growth of tourism activities and the rapid growth of demand for urbanization, land prices are increasing significantly and consequently the balance between the agriculture and urban sectors will shift toward the market demand and cost-effective investments.

Hence, spatial planning will enhance the ability of this region to cope with the existing and anticipated pressure from growing population, industrialization, energy projects, and the development of beachfront coastal resorts. Over and above, the anticipated impacts hazards, and risks of climate change either in the rising of sea level or the frequent occurrence of extreme events. Accordingly, it is deemed important to continuously evaluate different human activities in the study region as well as the early signals of climate change impacts, and to adopt sustainable (LULC) activities, such as careful monitoring of coastal tourism growth along the study area’s coastal strip, agriculture land conservation, and soil restoration. The effective implementation of the proposed plans for the development of the Mediterranean northwestern coast of Egypt has to consider the integration of the new tourism centers and agriculture sector to optimize their benefit through the strategy of agro-tourism.

To adverse the undesirable future outcomes related to (LULCCs) and perhaps any conflict in the study area, and to ensure the sustainability of the resource management of this area, an area-specific integrated coastal zone and resource management plan including conservation measures and the provision of livelihood alternatives that should be formulated and acceded within the national ICZM plan with the participation of the main stakeholders and beneficiaries.

Finally, the best practice of land management, and active participation of the stakeholders, and the local community should be enhanced to achieve this area’s sustainability and avoid any possible conflicts in land use in the future. These (LULCC) data are very useful for sustainable management and will support the decision-making process for the sustainable development of this area.

## Data Availability

The datasets generated during and/or analyzed during the current study are available from the corresponding author upon reasonable request.

## Declarations

All authors have read, understood, and have complied as applicable with the statement on “Ethical responsibilities of Authors” as found in the Instructions for Authors.
